# Optimizing Whole Brain Radiotherapy Treatment and Dose for Patients With Brain Metastases From Small Cell Lung Cancer

**DOI:** 10.3389/fonc.2021.726613

**Published:** 2021-10-25

**Authors:** Hanming Li, Wang Li, Chao Qi, Lu Zhou, Fengyun Wen, Yanli Qu, Hong Yu

**Affiliations:** ^1^ School of Graduate, Dalian Medical University, Dalian, China; ^2^ Department of Radiation Oncology, Cancer Hospital of China Medical University, Liaoning Cancer Hospital and Institute, Shenyang, China

**Keywords:** small cell lung cancer, brain metastases, whole brain radiotherapy, overall survival, biologically effective dose

## Abstract

**Purpose:**

This study aimed to evaluate the survival outcomes of whole brain radiotherapy (WBRT) compared to whole brain radiotherapy plus local radiation boost (WBRT + boost), and further identify whether higher biologically effective dose (BED) of WBRT + boost translates into a survival benefit in small cell lung cancer (SCLC) patients with brain metastasis (BM).

**Methods:**

SCLC patients with BM from January 1, 2012, to December 31, 2019, were retrospectively analyzed. Overall survival (OS) and intracranial progression-free survival (iPFS) were evaluated by the Kaplan–Meier method and compared by the log-rank test. Univariate and multivariate regression analyses of prognostic factors for OS were performed using Cox proportional hazards regression models. The cutoff value of BED was determined by the receiver operating characteristic (ROC) curve analysis.

**Results:**

Among the 180 eligible patients, 82 received WBRT + boost and 98 received WBRT. Both OS and iPFS in the WBRT + boost group were significantly superior to those in the WBRT group (median OS: 20 *vs*. 14 months, *p* = 0.011; median iPFS: 16 *vs*. 10 months, *p* = 0.003). At a cutoff value of 58.35 Gy in the WBRT + boost group, 52 for the high-BED (>58.35 Gy) group, 30 for the low-BED (≤58.35 Gy) group. High BED was significantly associated with improved OS and iPFS compared with low BED in the WBRT + boost group (median OS: 23 *vs*. 17 months, *p* = 0.002; median iPFS: 17 *vs*. 10 months, *p* = 0.002).

**Conclusions:**

Compared with WBRT alone, WBRT + boost improved OS and iPFS in SCLC patients with BM. High BED (>58.35 Gy) for WBRT + boost may be a reasonable consideration for SCLC patients with BM.

## Introduction

Small cell lung cancer (SCLC) is an aggressive form of lung cancer characterized by rapid dissemination and early metastasis (1, 2). Brain metastasis (BM) is the most common mode of metastasis in SCLC, which seriously affected the survival outcome (3). Up to 10% of patients are diagnosed with BM initially, and approximately 60% to 80% of patients will develop BM within 2 years after the initial diagnosis (4). For decades, whole brain radiotherapy (WBRT) has been the standard treatment for SCLC patients with BM (5). Recently, stereotactic radiosurgery (SRS) has been recommended increasingly for its high local control and low neurotoxicity, preferred for brain oligometastases (6–8). A large matched-cohort analysis, the FIRE-SCLC study, reported similar survival for upfront SRS versus WBRT for SCLC (9). However, this indication remains controversial in SCLC patients. Previous randomized clinical trials have suggested that WBRT combined with SRS could reduce intracranial recurrence rate (10). For patients with poor performance status, some retrospective studies have demonstrated that whole brain radiotherapy plus local radiation boost (WBRT + boost) could improve the intracranial control and prolong the survival time (11–13). Several retrospective studies suggested that high biologically effective dose (BED) for brain radiotherapy could improve survival among SCLC patients with BM (14, 15). This dose–response benefit suggests the value of evaluating BED in SCLC patients with BM. Brain radiotherapy remains controversial, and no consensus has been reached with regard to the optimal dose-escalation strategy in the management of SCLC patients with BM. Therefore, this study aimed to evaluate the survival benefits of WBRT + boost and WBRT in SCLC patients with BM and identify the dose–response benefit of BED in brain radiotherapy.

## Materials and Methods

### Patients

**Figure 1:**
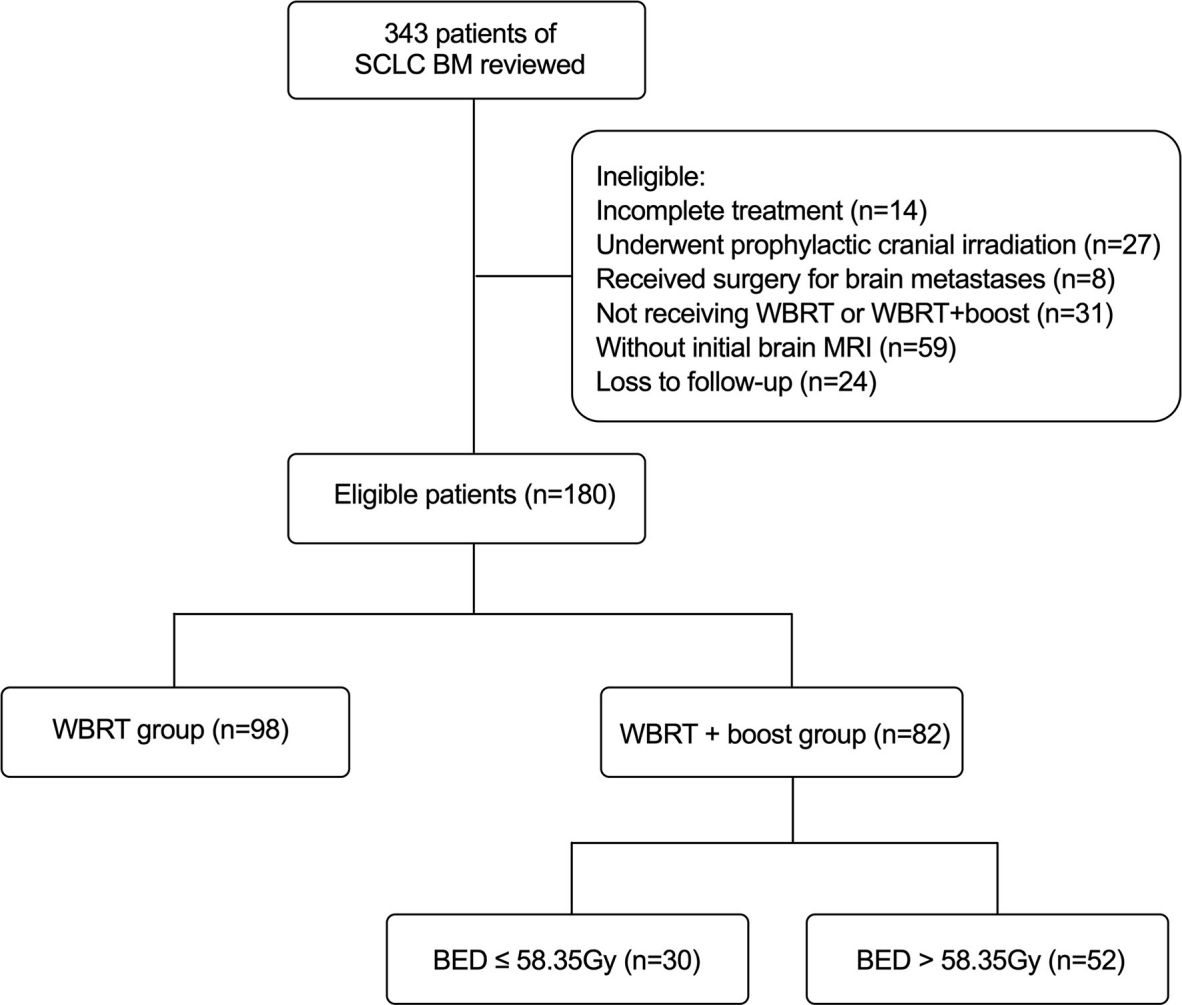
The flowchart of patient selection.

This was a single-center retrospective study. A total of 180 SCLC patients with BM in our institution between January 1, 2012, and December 31, 2019, were included. The eligible criteria for this study were as follows: pathologically identified SCLC and initial contrast-enhanced MRI identified brain metastases; completed treatment with corresponding follow-up information; and treated with WBRT or WBRT + boost for BM. Besides, routine brain MRI surveillance was performed on average every 3 months within the first 3 years after WBRT, and 6 months after 3 years. The exclusion criteria were as follows: without initial brain MRI; combined with other primary malignant tumors; incomplete treatment; underwent prophylactic cranial irradiation (PCI) or any surgery; and loss to follow-up. Overall survival (OS) was defined as the duration from the initial diagnosis of BM to death or the final follow-up (March 1, 2021). Intracranial progression-free survival (iPFS) was defined as the duration from the initial diagnosis of BM to the progression of intracranial metastases or death or the final follow-up (March 1, 2021). According to the brain radiotherapy, the 180 eligible patients were divided into two groups: the WBRT + boost group (*n* = 82) and the WBRT group (*n* = 98) (**Figure 1**). The Institutional Review Board of Cancer Hospital of China Medical University approved the study and the informed consent waiver.

### Treatment

**Figure 2:**
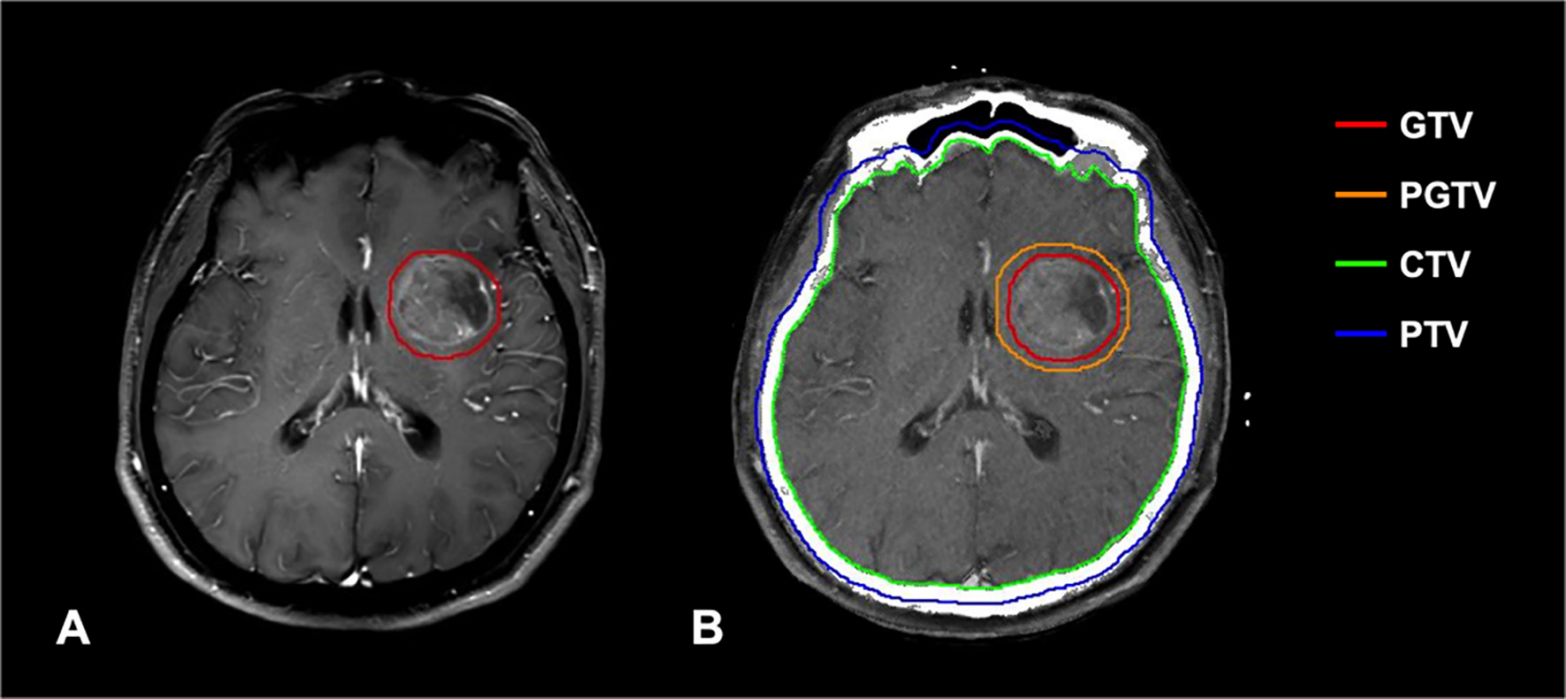
**(A)** T1-weighted contrast-enhanced MRI shows high signal intensity in the metastatic lesion. **(B)** Delineation of important RT volumes (GTV, PGTV, CTV, and PTV) was based on contrast-enhanced MRI.

Patients were immobilized with a head holder and a thermoplastic mask. A spiral CT (Philips Brilliance Big Bores CT) simulation scan was performed from the vertex through the upper cervical spine in the supine position with 3-mm slice thickness. All the scanned images were uploaded to the treatment planning system (Eclipse and ARIA, Varian Medical Systems Inc, Palo Alto, CA, USA), and then fused with contrast-enhanced MRI. Target volume delineation was based on the ICRU 52 and 62 recommendations. Clinical target volume (CTV) for WBRT was defined as the whole brain; a margin of 3 mm to CTV was used as planning target volume (PTV). Gross tumor volume (GTV) was delineated based on contrast-enhanced MRI sequences fused with planning CT scan; a margin of 3 mm to GTV was used as the planning gross target volume (PGTV) (**Figure 2**). Radiosensitive organs at risk (OARs) including lens, eyes, optic nerves, optic chiasm, pituitary gland, brainstem, and spinal cord were delineated. Brain radiotherapy was performed using three-dimensional conformal radiotherapy (3D-CRT) and intensity-modulated radiotherapy (IMRT). Local radiation boost to WBRT was delivered by sequential integrated boost (SEB) and simultaneous integrated boost (SIB) technique. The BED of BM was calculated based on a linear-quadratic model (BED = nd[1+d/(α/β)], *a*/β = 10 Gy) (16). Detailed dose and fractionation scheme were as follows: in the WBRT group, PTV: 30–40 Gy/10–20f (BED: 39–48 Gy) (5 fractions/week); in the WBRT + boost group, PTV: 30–40 Gy/10–20f (BED: 39–48 Gy), PGTV: 4–36 Gy/2–15 f (BED: 4.8–46.8 Gy) by SEB within 0–3 months after WBRT (5 fractions/week); PTV: 20–44 Gy/10–20f, PGTV: 25-52 Gy/10–20f (BED: 31.25–65.52 Gy) by SIB (5 fractions/week). Only one patient in the WBRT + boost group used an unconventional dose and fractionation scheme (PTV: 20 Gy/10f, PGTV: 25 Gy/10f) by SIB due to the poor performance with multiple extracranial metastases at the initial diagnosis of BM. The median cumulative BED of BM was 60.2 Gy (range 31.25–85.8 Gy) in the WBRT + boost group. All patients received over two cycles of platinum-based doublet chemotherapy before brain radiotherapy. In the course of treatment, dexamethasone and mannitol were given routinely to reduce intracranial pressure.

### Statistical Analysis

Clinical characteristics in categorical variables were calculated by Pearson’s Chi-square test and Fisher’s exact test. Receiver operating characteristic (ROC) curve was used to determine the cutoff value of BED in the WBRT + boost group. Kaplan–Meier survival analysis was performed for OS and iPFS in different groups. The log-rank test was used for the comparison of survival curves. Univariate and multivariate regression analyses of prognostic factors for OS and iPFS were performed using Cox proportional hazards regression models. *p* < 0.05 was considered statistically significant. All statistical analyses were performed using the SPSS Statistics Version 17.0 (SPSS Inc, Chicago, IL) and GraphPad Prism Version 9.0 (GraphPad Software, La Jolla, CA).

## Results

### Patient Characteristics

The baseline characteristics are presented in **Table 1** and were balanced between the groups. Among the 180 included patients, 98 patients underwent WBRT and 82 patients underwent WBRT + boost. The mean age of patients was 60 ± 8 years (range: 34–87 years) at the initial BM diagnosis; 42.2% (76/180) were under 60 years; 79.4% (143/180) of the patients were male.

**Table 1:** Baseline characteristics of 180 SCLC patients with BM.

Characteristics	Total	WBRT	WBRT + boost	*p*-value
	(*N* = 180)	(*n* = 98, 54.4%)	(*n* = 82, 45.6%)	
Age, years				0.053
<60	76 (42.2)	35 (35.7)	41 (50.0)	
≥60	104 (57.8)	63 (64.3)	41 (50.0)	
Sex				0.125
Female	37 (20.6)	16 (16.3)	21 (25.6)	
Male	143 (79.4)	82 (83.7)	61 (74.4)	
Smoking				0.252
No	94 (52.2)	55 (56.1)	39 (47.6)	
Yes	86 (47.8)	43 (43.9)	43 (52.4)	
KPS				0.497
≤80	84 (46.7)	48 (49.0)	36 (43.9)	
>80	96 (53.3)	50 (51.0)	46 (56.1)	
DS-GPA				0.544
≤2.0	79 (43.9)	41 (41.8)	38 (46.3)	
>2.0	101 (56.1)	57 (58.2)	44 (53.7)	
Number of BMs				0.144
1–5	138 (76.7)	71 (72.4)	67 (81.7)	
>5	42 (23.3)	27 (27.6)	15 (18.3)	
Maximum diameter of BM, cm				0.095
≤2.0	78 (43.3)	48 (49.0)	30 (36.6)	
>2.0	102 (56.7)	50 (51.0)	52 (63.4)	
Symptoms of BM				0.441
No	80 (44.4)	41 (41.8)	39 (47.6)	
Yes	100 (55.6)	57 (58.2)	43 (52.4)	
Extracranial metastasis				0.536
No	142 (78.9)	79 (80.6)	63 (76.8)	
Yes	38 (21.1)	19 (19.4)	19 (23.2)	
Radiation type				**0.001**
3D-CRT	115 (63.9)	79 (80.6)	36 (43.9)	
IMRT	65 (36.1)	19 (19.4)	46 (56.1)	

WBRT, whole brain radiotherapy; WBRT + boost, whole brain radiotherapy plus local radiation boost; KPS, Karnofsky Performance Status; DS-GPA, diagnosis-specific graded prognostic assessment; BM, brain metastasis.

The bold values inidicate p-value is less than 0.05.

### Survival Outcomes

**Figure 3:**
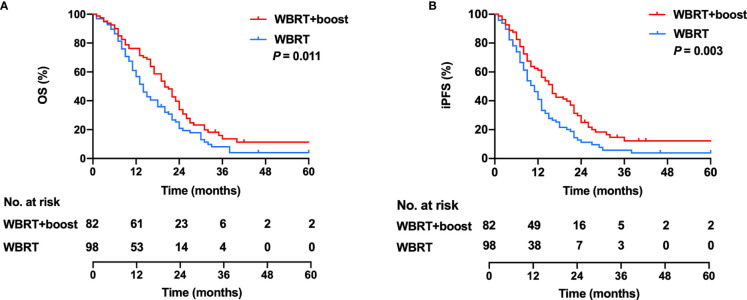
Kaplan–Meier curves for OS **(A)** and iPFS **(B)** in the WBRT and the WBRT + boost group.

All patients were closely followed up, and the median follow-up time was 40 months (range, 1–96 months). The median OS was 17 months, with 1- and 2-year OS rates of 63.3% and 20.6%. In the WBRT + boost group, the median OS was 20 months, with 1- and 2-year OS rates of 74.3% and 28.0%. In the WBRT group, the median OS was 14 months, with 1- and 2-year OS rates of 54.1% and 14.3%. Significant differences in OS were observed between the WBRT + boost and the WBRT groups (*p* = 0.011, **Figure 3A**).

The median iPFS was 12 months, with 1- and 2-year iPFS rates of 48.3% and 12.8%. In the WBRT + boost group, the median iPFS was 16 months, with 1- and 2-year iPFS rates of 59.8% and 19.5%. In the WBRT group, the median iPFS was 10 months, with 1- and 2-year iPFS rates of 38.8% and 7.1%. Significant differences in iPFS were observed between WBRT + boost and WBRT groups (*p* = 0.003, **Figure 3B**).

### Univariate and Multivariate Cox Regression Analyses for OS and iPFS

Univariate Cox regression analysis showed that age (*p* = 0.038), number of BMs (*p* = 0.013), and radiotherapy treatment (*p* = 0.014) were remarkable prognostic indicators associated with OS. Multivariate Cox regression analysis showed that WBRT + boost (HR = 0.69, 95% CI: 0.50–0.96, *p* = 0.028) was independently associated with better OS, while number of BMs > 5 (HR = 1.49, 95% CI: 1.02–2.17, *p* = 0.039) was correlated with worse OS (**Table 2**). Additionally, age, number of BMs, and radiotherapy treatment were also significantly associated with iPFS in the univariate analysis. Multivariate Cox regression analysis showed that WBRT + boost (HR = 0.63, 95% CI: 0.46–0.88, *p* = 0.006) was independently associated with better iPFS (**Table 3**).

**Table 2:** Univariate and multivariate analyses of factors influencing OS of 180 SCLC patients with BM.

Variables	Univariate analysis		Multivariate analysis	
	HR (95% CI)	*p*-value	HR (95% CI)	*p*-value
Age, years				
<60/≥60	1.42 (1.02–1.97)	**0.038**	1.40 (0.96–1.87)	0.085
Sex				
Female/Male	1.28 (0.84–1.95)	0.259		
Smoking				
No/Yes	1.13 (0.82–1.57)	0.453		
KPS				
≤80/>80	0.89 (0.65–1.23)	0.486		
DS-GPA				
≤2.0/>2.0	0.78 (0.56–1.08)	0.140		
Number of BMs				
1–5/> 5	1.60 (1.10–2.33)	**0.013**	1.49 (1.02–2.17)	**0.039**
Maximum diameter of BM, cm				
≤2.0/>2.0	1.21 (0.88–1.68)	0.245		
Symptoms of BM				
No/Yes	0.93 (0.69–1.26)	0.651		
Extracranial metastasis				
No/Yes	1.13 (0.76–1.68)	0.548		
Treatment				
WBRT/WBRT+boost	0.63 (0.48–0.92)	**0.014**	0.69 (0.50–0.96)	**0.028**
Radiation type				
3D-CRT/IMRT	0.79 (0.56–1.12)	0.183		

OS, overall survival; HR, hazard ratio; CI, confidence interval; WBRT, whole brain radiotherapy; WBRT + boost, whole brain radiotherapy plus local radiation boost; KPS, Karnofsky Performance Status; BM, brain metastasis; DS-GPA, diagnosis-specific graded prognostic assessment.

The bold values inidicate p-value is less than 0.05.

**Table 3:** Univariate and multivariate analyses of factors influencing iPFS of 180 SCLC patients with BM.

Variables	Univariate analysis		Multivariate analysis	
	HR (95% CI)	*p*-value	HR (95% CI)	*p*-value
Age, years				
<60/≥60	1.45 (1.04–2.07)	**0.026**	1.35 (0.97–1.88)	0.078
Sex				
Female/Male	1.53 (1.00–2.33)	0.051		
Smoking				
No/Yes	1.00 (0.73–1.38)	0.981		
KPS				
≤80/>80	0.81 (0.59–1.12)	0.199		
DS-GPA				
≤2.0/>2.0	0.77 (0.56–1.06)	0.110		
Number of BMs				
1–5/>5	1.53 (1.06–2.20)	**0.024**	1.43 (0.99–2.08)	0.060
Maximum diameter of BM, cm				
≤2.0/> 2.0	1.12 (0.81–1.54)	0.485		
Symptoms of BM				
No/Yes	0.92 (0.69–1.26)	0.578		
Extracranial metastasis				
No/Yes	1.07 (0.72–1.58)	0.740		
Treatment				
WBRT/WBRT + boost	0.62 (0.45–0.86)	**0.004**	0.63 (0.46–0.88)	**0.006**
Radiation type				
3D-CRT/IMRT	0.84 (0.60–1.18)	0.315		

iPFS, intracranial progression-free survival; HR, hazard ratio; CI, confidence interval; WBRT, whole brain radiotherapy; WBRT + boost, whole brain radiotherapy plus local radiation boost; KPS, Karnofsky Performance Status; BM, brain metastasis; DS-GPA, diagnosis-specific graded prognostic assessment.

The bold values inidicate p-value is less than 0.05.

### Patterns of Progression

Overall, 90 patients developed tumor progression. Patterns of the first site of progression in the WBRT and WBRT + boost groups were, respectively, intracranial in 19 (33.9%) and 11 (32.4%), and extracranial in 37 (66.1%) and 23 (67.6%) (**Table 4**).

**Table 4:** Patterns of failure in 90 patients after WBRT or WBRT + boost.

Pattern of progression	Total	WBRT	WBRT + boost
	(*N* = 90)	(*n* = 56, 62.2%)	(*n* = 34, 37.8%)
Intracranial	30 (33.3)	19 (33.9)	11 (32.4)
Extracranial	60 (66.7)	37 (66.1)	23 (67.6)

WBRT, whole brain radiotherapy; WBRT + boost, whole brain radiotherapy plus local radiation boost.

### BED-Based Dose Escalation in the WBRT + Boost Group

**Figure 4:**
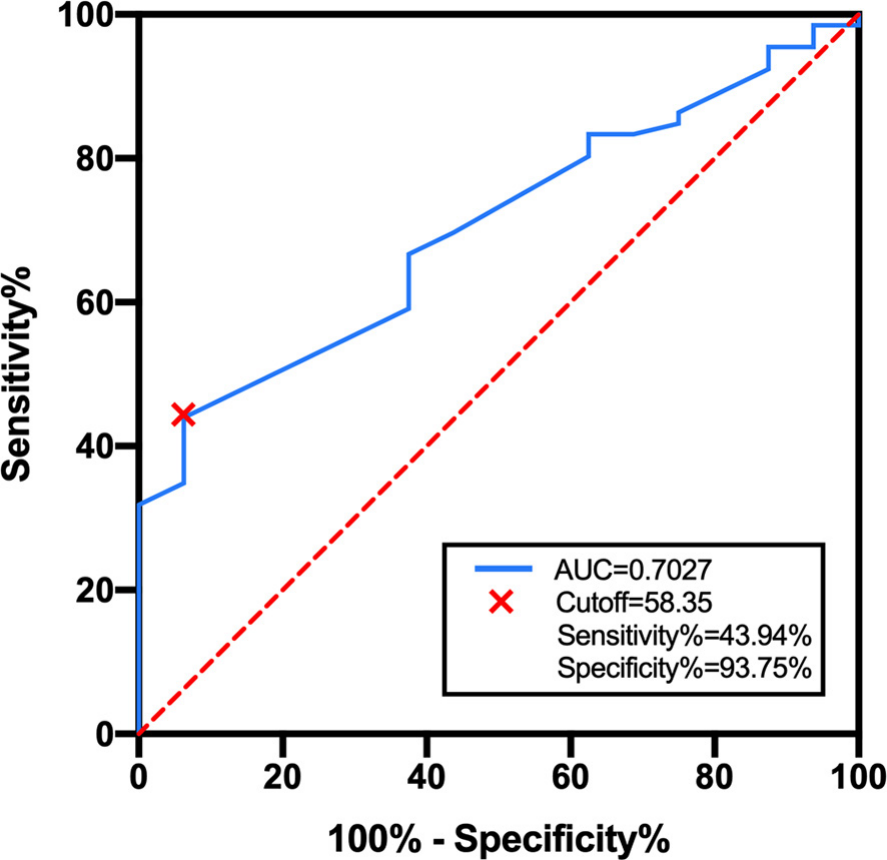
ROC curve for BED in the WBRT + boost group. According to the Youden index of 0.376, 58.35 Gy was taken as the optimal cutoff value with a sensitivity of 44.94% and a specificity of 93.75%. Area under the curve was 0.703 (95% CI: 0.589–0.825, *p* = 0.012).

**Figure 5:**
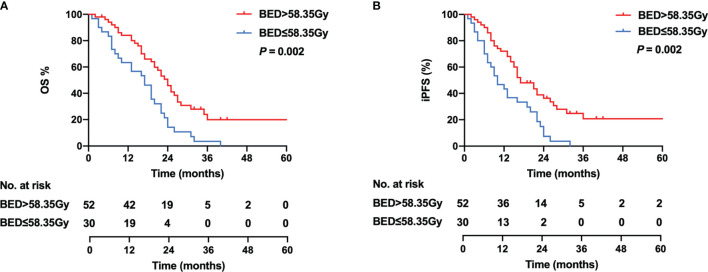
Kaplan–Meier curves for OS **(A)** and iPFS **(B)** in the high-BED group (BED > 58.35 Gy) and the low-BED group (BED ≤ 58.35 Gy).

As there is no consensus on the optimal dose escalation of radiotherapy in SCLC patients with BM, we investigated the relationship between BED-based dose escalation and survival outcomes in the WBRT + boost group. According to the Youden index of 0.376, 58.35 Gy was taken as the optimal cutoff value of BED by the ROC analysis (**Figure 4**). Of 82 patients, BED > 58.35 Gy (52 patients, 63.4%) was defined as the high-BED group, and BED ≤ 58.35 Gy (30 patients, 36.6%) was defined as the low-BED group. Baseline characteristics were comparable between the two groups (**Table S1**). Significant differences in OS were observed between the high-BED and the low-BED group (23 *vs*. 17 months; *p* = 0.002), with 1- and 2-year OS rates of 80.8% and 36.5% in the high-BED group, and 63.3% and 13.3% in the low-BED group (**Figure 5A**). Similar results in iPFS were observed between the high-BED and the low-BED group (17 *vs*. 10 months; *p* = 0.002), with 1- and 2-year OS rates of 69.2% and 26.9% in the high-BED group, and 43.3% and 6.7% in the low-BED group (**Figure 5B**).

## Discussion

SCLC patients with BM have a poor prognosis with a median OS ranging between 2 and 14 months (17). The radiotherapy treatment and optimal dose for patients with SCLC BM remain controversial, and no consequence has been reached. Our study showed that compared with WBRT alone, the OS and iPFS of patients who received WBRT + boost were significantly improved. High BED (>58.35 Gy) for WBRT + boost may further improve survival.

WBRT can alleviate the neurological symptoms, remaining a commonly used treatment for SCLC patients with BM. Considering the tolerable dose of normal brain tissue, WBRT is limited in intracranial control. Approximately 33% of BM patients after WBRT were uncontrolled, and 50% of patients died from the progression of intracranial lesions (18). Recently, WBRT combined with local boost radiotherapy has gradually shown advantages in the management of patients with BM. SIB or SEB to WBRT on brain metastases can further improve intracranial control and prolong survival time. The RTOG 9508 randomized clinical trial found that compared with WBRT alone, WBRT combined with SRS significantly improved the OS of patients with single BM (6.5 *vs*. 4.9 months, *p* = 0.039) (10). However, due to the high technical requirement, SRS is not available for routine treatment in most institutions. In recent years, many studies have demonstrated that WBRT combined with SIB or SEB is associated with better OS than WBRT alone (19, 20). Dobi et al. analyzed 468 patients with BMs from various primary tumors, and they found that WBRT + boost increased OS than WBRT alone (6.5 *vs*. 3.3 months, *p* < 0.001) (11). Of note, only 65 SCLC patients were included in this study. Sun et al. analyzed 82 patients with SCLC BM, and WBRT + boost significantly improved OS than WBRT alone (*n* = 49) (13.4 *vs*. 9.6 months, *p* = 0.004) (12). Another study analyzed 263 SCLC BM patients, and they found that WBRT + boost resulted in longer OS than WBRT (17.9 *vs*. 8.7 months, *p* < 0.001) (13). Our findings were consistent with the previous studies. We investigated that WBRT + boost could generate better survival outcomes, with 1- and 2-year OS rates of 74.4% and 28.0%. WBRT + boost may be a preferred strategy for SCLC patients with BM. Although WBRT alone is highly effective to prevent metastatic spread in the brain, the local control on larger or single lesion is limited due to the lower dose. While the SIB or SEB to WBRT on metastatic lesions can increase the local control rate and further improve survival.

There is no consensus on the dose–response benefit of local radiation boost, and it is unclear whether increasing the dose of local radiation boost will further translate into a survival benefit. According to the L-Q linear model, different dose-fractionation schemes lead to different BEDs (16). A retrospective study has shown that high-dose (tBED > 50 Gy) thoracic radiotherapy can improve survival in extensive-stage SCLC patients, compared with low-dose (tBED ≤ 50 Gy) thoracic radiotherapy (21). The study suggested the feasibility and the potential prognostic value of evaluating BED in the management of SCLC patients who underwent radiotherapy. A meta-analysis showed that for patients with BM who received fractional SRS therapy, when the BED was 40, 50, and 60 Gy, 1-year intracranial local control rates were 73%, 78%, and 84%, respectively, and 2-year intracranial local control rates were 62%, 69%, and 81%, respectively (22). The intracranial local control was improved along with increasing BED, showing the benefit of BED-based dose–response in patients with BM. A multi-center retrospective study reported that a total dose > 39 Gy (BED > 50.7 Gy) was associated with improved OS in patients with BM receiving WBRT + boost (23.3 months *vs*. 8.2 months, *p* < 0.01) (15). Interestingly, the medical center was significantly correlated with improved survival in the univariate analysis. One center always administered 36 Gy/12f WBRT combined with sequential local boost dose of 18 Gy/9f (BED = 64.8 Gy), while most other centers used 30 Gy/10f WBRT combined with a local radiation boost dose of 9 Gy/3f (BED = 50.7 Gy). The magnitude of the nearly >28% increase in BED might be expected to result in an increase in the local control for BM patients, and further improved OS. An appropriate increase in BED may bring survival benefits to patients treated with WBRT + boost. Another retrospective study included 250 SCLC BM patients, suggesting that the use of BED > 47.4 Gy brain radiotherapy can improve OS and iPFS (14). In this study, 208 patients received WBRT, and 42 patients received WBRT + boost, but the study failed to address the relationship between brain radiotherapy treatment and BED. We believed that the difference in radiotherapy treatment has a more significant impact on BED compared with different dose-fractionation scheme. In our study, the WBRT group and WBRT + boost group received a quite similar BED on BMs (39–48 Gy *vs*. 39–53.68 Gy). Therefore, excluding the impact of radiotherapy treatment on BED, we further conducted analyses to investigate the distribution of BED and its contribution to survival outcomes in the WBRT + boost group. Our study indicated that in patients treated with BED > 58.35 Gy for WBRT + boost, the median OS and 1-year OS rate were 23 months and 80.8%, respectively. The survival of using BED > 58.35 Gy for brain radiotherapy was better than that of using BED > 47.4 Gy previously reported, in which the median OS and 1-year survival rate were 17.5 months and 71.1%, respectively (14).

We also analyzed potential prognostic factors affecting OS and iPFS in SCLC patients receiving brain radiotherapy for BM. As previously reported, several prognostic factors have been identified, including age, KPS, extracranial metastases status, number of BMs, the maximum diameter of BM, and symptoms of BM (12, 13). Our study revealed that the number of BMs and treatment were independent prognostic factors for OS, while it was treatment for iPFS. Extracranial metastases status at the time of initial diagnosis of BM, as mentioned above, was usually considered as a prognostic factor, but unexpectedly, we did not prove that it is one of the predictors in both univariate and multivariate Cox regression analyses. This negative result could be related to the different sites of metastases. Previous studies have suggested that liver metastasis was associated with a poor prognosis (23, 24). In this study, only 21.1% (38/180) of patients had extracranial metastases at the time of the initial diagnosis of BM, and 34.2% (15/38) of them had liver metastasis. Therefore, these reasons may impact the results, and the conclusion needs to be further confirmed by a large sample study. There is no consistent conclusion on the prognostic value of the number of BMs. Ni et al. showed that 1–3 BMs were independently associated with improved OS, consistent with our findings (13). However, Bernhardt et al. found that numbers of BM were not an independent prognostic factor for SCLC patients with BM (25). The controversy of the results may be due to the total volume of BMs. Several studies revealed that compared with the number of BMs, the total volume of BMs might be a more important factor (26, 27). Validation of the value of the total volume of BMs ought to be investigated and confirmed in larger studies.

In terms of the first site of progression after brain radiotherapy, extracranial disease progression is the main failure pattern. In this study, few patients developed intracranial progression, while more patients developed extracranial progression. WBRT and WBRT + boost can improve the local control rate of intracranial lesions and reduce the possibility of intracranial or neurologic death. Therefore, how to improve intracranial control while reducing distant metastasis will be the future direction. Recently, phase III clinical IMpower133 and CASPIAN trials showed that immunotherapy has become a component of standard therapy in the frontline setting for ES-SCLC (28, 29). Retrospective studies have shown that local treatment such as SRS combined with systemic chemotherapy or immunotherapy is considered the therapeutic option in SCLC patients with BM (30). Moreover, if future research on immunotherapy and other systemic agents can demonstrate enhanced CNS activity in SCLC, such developments will result in changing patterns of SCLC systemic therapy strategies for addressing CNS micrometastases and extracranial progression. In the era of immunotherapy, it is necessary to re-evaluate the addition of WBRT + boost, which needs to be verified by prospective randomized clinical trials.

Meanwhile, radiation-related neurotoxicity caused by WBRT cannot be ignored. The hippocampus is sensitive to radiation and regarded as a potential contributing cause for neurocognitive deficits after WBRT. RTOG 0933 trial demonstrated that hippocampal avoidance WBRT (HA-WBRT) was associated with improved memory and QoL (31). Hippocampal avoidance during WBRT + boost may further improve intracranial control and reduce cognitive decline, which may be a feasible strategy for SCLC patients. A multicenter phase II HIPPORAD trial is ongoing to evaluate the potential of hippocampal-sparing whole brain irradiation with simultaneous integrated boost (HSIB-WBRT) to prevent neurocognitive adverse effects (32).

It should be noted that limitations in our research exist. First, this is a retrospective study, and consequent patient heterogeneity may have biased the results. Second, we failed to evaluate dose escalation-related neurotoxicity and the overall volume of BM. Third, although compared with prior studies, this is a relatively large sample size for SCLC patients with BM, additional patients may be needed to determine which patients may be most likely to benefit from WBRT + boost. Fourth, SRS has been recommended increasingly for its high local control and low neurotoxicity. SCLC patients with BM were treated with WBRT or WBRT + boost at our department, in lack of SRS availability before 2020. Our study provides valuable conclusions for many institutions who did not implement SRS yet. In this study, the large number of SCLC patients with BM and the two treatment approaches were clearly defined. Conclusion could be drawn from this analysis on the applicability of WBRT + boost for SCLC patients with BM. Furthermore, higher BED of WBRT + boost seems to yield clinical benefit.

## Conclusions

Our study showed a significant improvement in survival outcomes by introducing WBRT + boost. High BED for WBRT + boost may be a preferred strategy for SCLC patients with BM. Further validation in large randomized controlled trials is required to facilitate the individual options and minimize neurotoxicities when conducting WBRT + boost with a BED of at least 58.35 Gy.

## Data Availability Statement

The raw data supporting the conclusions of this article will be made available by the authors, without undue reservation.

## Ethics Statement

The Institutional Review Board of Cancer Hospital of China Medical University approved this retrospective study and a waiver of patient’s informed consent was granted due to the retrospective nature of this study.

## Author Contributions

HL and HY designed the research. HL and CQ performed data acquisition. HL and WL performed the statistical analysis. HL, LZ, YQ, and FW drafted the manuscript. All authors contributed to the article and approved the submitted version.

## Conflict of Interest

The authors declare that the research was conducted in the absence of any commercial or financial relationships that could be construed as a potential conflict of interest.

## Publisher’s Note

All claims expressed in this article are solely those of the authors and do not necessarily represent those of their affiliated organizations, or those of the publisher, the editors and the reviewers. Any product that may be evaluated in this article, or claim that may be made by its manufacturer, is not guaranteed or endorsed by the publisher.
