# Supplementary material for: Optimizing Whole Brain Radiotherapy Treatment and Dose for Patients With Brain Metastases From Small Cell Lung Cancer
Source: Front Oncol. 2021 Oct 25;11:726613. doi: 10.3389/fonc.2021.726613 (PMC8573246; doi:10.3389/fonc.2021.726613)
Supplement: Supplementary file 1 [file DataSheet_1.docx]

Supplementary Material

# Supplementary Table 1 Baseline characteristics of 82 patients in the WBRT+boost group.

| **Characteristics** | **Total** | **BED ≤ 58.35Gy** | **BED > 58.35Gy** | ***P* value** |
| --- | --- | --- | --- | --- |
|  | **(N=82)** | **(n=30 36.6%)** | **(n=52 63.4%)** |  |
| Age year, y |  |  |  | 0.067 |
| <60 | 41 (50.0) | 11 (36.7) | 30 (57.7) |  |
| ≥60 | 41 (50.0) | 19 (63.3) | 22 (42.3) |  |
| Sex |  |  |  | 0.377 |
| Female | 21 (25.6) | 6 (20.0) | 15 (28.8) |  |
| Male | 61 (74.4) | 24 (80.0) | 37 (71.2) |  |
| Smoking |  |  |  | 0.087 |
| No | 39 (47.6) | 18 (60.0) | 21 (40.4) |  |
| Yes | 43 (52.4) | 12 (40.0) | 31 (59.6) |  |
| KPS |  |  |  | 0.937 |
| ≤80 | 36 (43.9) | 13 (43.3) | 23 (44.2) |  |
| >80 | 46 (56.1) | 17 (56.7) | 29 (55.8) |  |
| DS-GPA |  |  |  | 0.060 |
| ≤2.0 | 38 (46.3) | 18 (60.0) | 20 (38.5) |  |
| >2.0 | 44 (53.7) | 12 (40.0) | 32 (61.5) |  |
| Number of BMs |  |  |  | 0.772 |
| 1-5 | 67 (81.7) | 25 (83.3) | 42 (80.8) |  |
| >5 | 15 (18.3) | 5 (16.7) | 10 (19.2) |  |
| Maximum diameter of BM, cm |  |  |  | 0.991 |
| ≤2.0 | 30 (36.6) | 11 (36.7) | 19 (36.5) |  |
| >2.0 | 52 (63.4) | 19 (63.3) | 33 (63.5) |  |
| Symptoms of BM |  |  |  | 0.831 |
| No | 39 (47.6) | 14 (46.7) | 25 (48.1) |  |
| Yes | 43 (52.4) | 16 (53.3) | 27 (51.9) |  |
| Extracranial metastasis |  |  |  | 0.226 |
| No | 63 (76.8) | 21 (70.0) | 42 (80.8) |  |
| Yes | 19 (23.2) | 9 (30.0) | 10 (19.2) |  |

# **
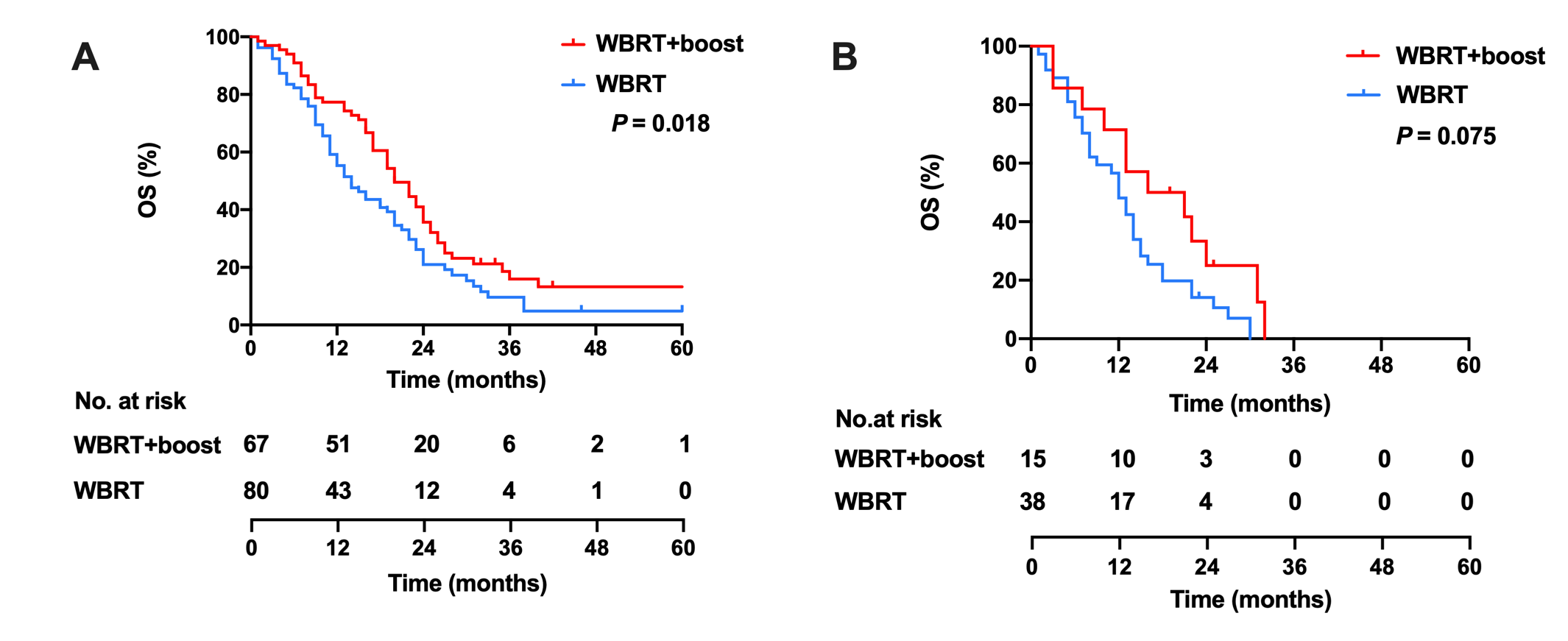
**

**
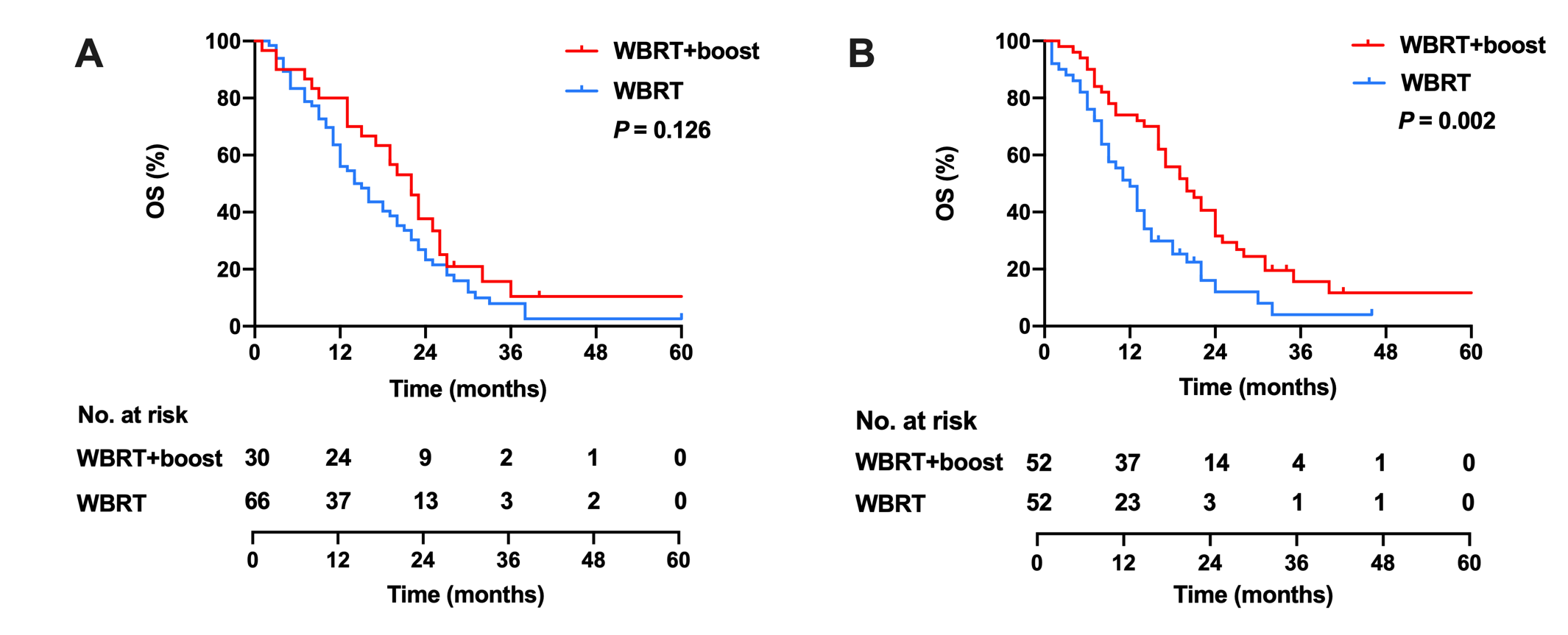
**
